# High-quality genome assembly of *Verticillium dahliae* VD991 allows for screening and validation of pathogenic genes

**DOI:** 10.3389/fmicb.2023.1177078

**Published:** 2023-05-31

**Authors:** Jiaxiang Yang, Lisen Liu, Lan Yang, Renju Liu, Chenxu Gao, Wei Hu, Qingdi Yan, Zhaoen Yang, Liqiang Fan

**Affiliations:** ^1^Zhengzhou Research Base, State Key Laboratory of Cotton Biology, School of Agricultural Sciences, Zhengzhou University, Zhengzhou, China; ^2^State Key Laboratory of Cotton Biology, Institute of Cotton Research of the Chinese Academy of Agricultural Sciences, Anyang, China; ^3^Zhengzhou Research Base, State Key Laboratory of Cotton Biology, Zhengzhou University, Zhengzhou, China

**Keywords:** *Verticillium dahliae*, genome assembly, cotton, pathogenic genes, WGCNA

## Abstract

*Verticillium dahliae* (*V. dahliae*) is a notorious soil-borne pathogen causing Verticillium wilt in more than 400 dicotyledonous plants, including a wide range of economically important crops, such as cotton, tomato, lettuce, potato, and romaine lettuce, which can result in extensive economic losses. In the last decade, several studies have been conducted on the physiological and molecular mechanisms of plant resistance to *V. dahliae*. However, the lack of a complete genome sequence with a high-quality assembly and complete genomic annotations for *V. dahliae* has limited these studies. In this study, we produced a full genomic assembly for *V. dahliae* VD991 using Nanopore sequencing technology, consisting of 35.77 Mb across eight pseudochromosomes and with a GC content of 53.41%. Analysis of the genome completeness assessment (BUSCO alignment: 98.62%; Illumina reads alignment: 99.17%) indicated that our efforts resulted in a nearly complete and high-quality genomic assembly. We selected 25 species closely related to *V. dahliae* for evolutionary analysis, confirming the evolutionary relationship between *V. dahliae* and related species, and the identification of a possible whole genome duplication event in *V. dahliae*. The interaction between cotton and *V. dahliae* was investigated by transcriptome sequencing resulting in the identification of many genes and pathways associated with cotton disease resistance and *V. dahliae* pathogenesis. These results will provide new insights into the pathogenic mechanisms of *V. dahliae* and contribute to the cultivation of cotton varieties resistant to Verticillium wilt.

## Introduction

**FIGURE 1:**
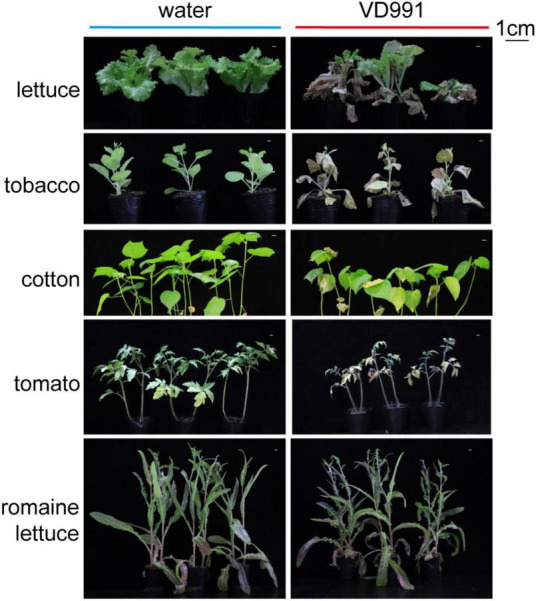
Phenotypes of five economic crops (lettuce, tobacco, cotton, tomato, and romaine lettuce) after inoculation with *Verticillium dahliae* VD991.

*Verticillium dahliae* (*V. dahliae*), a soil-borne fungus, can cause severe damage to many economically important crops (Klosterman et al., 2009), such as lettuce, tobacco, cotton, tomato, and romaine lettuce (Figure 1). Due to its unique microsclerotia structure, *V. dahliae* can survive in extreme temperatures from 80°C to −30°C and can survive in non-ideal conditions for more than 10 years (Fradin and Thomma, 2006; Inderbitzin and Subbarao, 2014). More worryingly, *V. dahliae* has a high degree of genetic diversity and widely varying pathogenicity and can co-evolve with its hosts to produce new and highly pathogenic physiological strains (Atallah et al., 2010; Song et al., 2020). Although there exist many control strategies for *V. dahliae*, including crop rotation, soil fumigation, biological controls, and chemical sterilization (Acharya et al., 2020; Ingram et al., 2020; Zhang Y. L. et al., 2021), the disease caused by *V. dahliae* still results in significant crop yield reductions and even cases of total loss each year (Zhang et al., 2023). Breeding *V. dahliae*-resistant varieties is a promising strategy for the effective control of *V. dahliae* infection.

The genomes of *V. dahliae* play an important role in studying the pathogenicity of *V. dahliae* and in revealing the interaction between the pathogen and its host. To date, a total of 43 different versions of the *V. dahliae* genome have been published,^1^ with genome assembly sizes ranging from 31.97 to 40.17 Mb and GC contents ranging from 53.20 to 56.4%. A total of 15 *V. dahliae* genomes have been assembled to the contig level, 19 *V. dahliae* genomes have been assembled to the scaffold level, and only 4 *V. dahliae* genomes (Getta, Gwydir1A3, VDLs.17, and VDJR2) (Klosterman et al., 2011; de Jonge et al., 2013) have been assembled to the chromosome level. The Getta and Gwydir1A3 genomes were assembled using Illumina HiSeq data, and the VDLs.17 and VDJR2 genomes were assembled using PacBio data, all of which were assembled into eight chromosomes. Unfortunately, these four genomes do not provide gene annotation results, which greatly limits the scope of the investigation into *V. dahliae* pathogenicity and the breeding of crop varieties resistant to *V. dahliae* infection (Supplementary Table 1).

Cotton is one of the most economically important crops in the world, primarily as a key source of fiber and oil (Rong et al., 2004; Ma et al., 2018; Yang et al., 2023). However, cotton is also one of the most common hosts for *V. dahliae*, which can cause great economic loss by reducing cotton yields (Yang et al., 2015; Song et al., 2021). To date, transcriptome sequencing has been used extensively to study mechanisms of resistance in cotton to *V. dahliae* infection. For example, Zhu et al. (2021) used transcriptomic analysis to reveal the gene regulatory network of resistance by comparing the resistant variant L38 with the susceptible variety J1 after *V. dahliae* infection. Xiong et al. (2021) showed that the *GhGDH2* gene regulates cotton resistance to Verticillium wilt through the JA and SA signaling pathways. Transcriptomic studies to date have mainly focused on changes in gene expression levels of cotton before and after inoculation with *V. dahliae*, further identifying disease-resistance genes and pathways, however, there are no studies on gene interactions between cotton and *V. dahliae*.

In this study, we performed whole-genome Nanopore sequencing of *V. dahliae* and assembled a high-quality reference genome. Gene prediction and functional annotation of the *V. dahliae* genome were carried out using multiple databases. The secreted proteins and effector proteins were also predicted because they were important pathogenic factors of *V. dahliae*. In addition, we obtained transcriptomic data for both cotton and *V. dahliae* by sequencing cotton root tips after inoculation with *V. dahliae* to investigate interacting genes and pathways.

## Materials and methods

### DNA extraction and genome sequencing

Strain VD991 of *V. dahliae* was grown on potato dextrose agar (PDA) until sufficient spores were produced for sample collection. DNA was extracted from the spores using a cetyl-trimethyl ammonium bromide (CTAB) method (Biel and Parrish, 1986; Wang et al., 2022). The DNA obtained was then quality tested (Nanodrop and 0.35% agarose gel electrophoresis) and quantified (Qubit). Large fragments of genomic DNA were recovered via the BluePippin automatic nucleic acid recovery system, and DNA libraries were prepared using the SQK-LSK109 Ligation Sequencing Kit. Sequencing was performed using the PromethION Flow Cell (R9 Version) (Oxford Nanopore Technologies) (Deamer et al., 2016; Lu et al., 2016).

### Genome assembly

The assembly of the *V. dahliae* VD991 was carried out using the following process: longer reads and high-quality reads were extracted using Filtlong v0.2.0^2^; the filtered Nanopore reads were assembled using NECAT v0.01 (Chen et al., 2021); quality control of Illumina short reads was conducted using Trimmomatic v0.30 (Bolger et al., 2014); the reads obtained were used for polishing of the sequences assembled from Nanopore reads; six rounds of assembly polishing were carried out on Illumina reads using Pilon v1.23 (Walker et al., 2014) to correct base-calling and insertion/deletion errors. Hi-C fragment libraries were constructed and Illumina HiSeq sequencing was performed. Clean reads were mapped to the *V. dahliae* genome using BWA v0.7.9 (Li and Durbin, 2009). Paired-end reads were mapped to the genome separately and filtered, followed by the collection of unique, mapped paired-end reads using HiC-Pro v2.10 (Servant et al., 2015). The order and direction of scaffolds/contigs were clustered into super scaffolds using LACHESIS, based on the relationships among valid reads. Finally, the data were assembled onto eight chromosomes. By aligning next-generation sequencing data for *V. dahliae* VD991 against the fully assembled genome, genome quality was assessed based on the percentage and coverage of mapped reads. In addition, the BUSCO (Benchmarking Universal Single-Copy Orthologs) method (based on 290 conserved core genes for fungi) was used to further assess assembly completeness and quality (Simao et al., 2015).

### Genome component analysis

As repetitive sequences tend to be poorly conserved among species, a species-specific repeat database was developed for *V. dahliae* VD991 using *de novo* and structural prediction as implemented in LTR_FINDER v1.05 (Xu and Wang, 2007), MITE-Hunter (Han and Wessler, 2010), PILER-DF v2.4 (Smith et al., 2007), and RepeatScout v1.0.5 (Price et al., 2005); PASTEClassifier v2.0 (Wicker et al., 2007) was then used to classify the repeated elements in the database. The newly constructed database was merged with the Repbase database (Bao et al., 2015), and repeated sequences were predicted for the *V. dahliae* VD991 genome using RepeatMasker v4.0.6 (Tarailo-Graovac and Chen, 2009).

Prediction of gene structure was performed using *de novo* prediction, homologous protein-based prediction, and transcriptome-based prediction. *De novo* prediction was conducted using Augustus v2.4 (Stanke and Waack, 2003), GeneID v1.4 (Blanco et al., 2007), Genscan (Burge and Karlin, 1997), GlimmerHMM v3.0.4 (Majoros et al., 2004), and SNAP v2006-07-28 (Korf, 2004), while homologous protein-based prediction was implemented in GeMoMa v1.3.1 (Keilwagen et al., 2016). The transcriptomic data were assembled using Hisat2 v2.0.4 (Pertea et al., 2016) and Stringtie v1.2.3 (Pertea et al., 2016), and unigene sequences predicted using PASA v2.0.2 (Campbell et al., 2006) and TransDecoder v2.0 (Tian et al., 2018). Finally, EVM v1.1.1 (Haas et al., 2008) was used to integrate the results from all three prediction methods.

Non-coding RNAs are RNAs that do not encode proteins, including RNAs with known functions such as microRNAs, rRNAs, and tRNAs. Using known structural characteristics of non-coding RNAs, tRNAs were predicted for the *V. dahliae* genome using tRNAscan-SE (Lowe and Eddy, 1997); rRNAs and other ncRNAs (i.e., not rRNAs and tRNAs) were predicted using Infernal v1.1 (Nawrocki and Eddy, 2013) and the Rfam database (Nawrocki et al., 2015).

Pseudogenes are similar in sequence to functional genes, but have become nonfunctional due to mutations such as insertions and deletions. To identify homologous gene sequences in the *V. dahliae* VD991 genome, predicted protein sequences were compared with protein sequences from the Swiss-Prot database using GenBlastA v1.0.4 (She et al., 2009). To identify pseudogenes, GeneWise (Birney et al., 2004) was used to find premature stop codons and frameshift mutations in known gene sequences.

### Genome annotation

The predicted gene sequences were aligned to functional databases [e.g., KEGG (Kanehisa et al., 2004), KOG (Tatusov et al., 2000), Nr (Deng et al., 2006), Swiss-Prot (Boeckmann et al., 2003) and TrEMBL (Boeckmann et al., 2003)] using BLAST v2.2.26 (Altschul et al., 1997) to annotate functional genes. Based on the Nr database annotation results, gene ontology (GO) annotation was performed using Blast2GO v5.2.5 (Conesa et al., 2005) and the GO database (Ashburner et al., 2000), while Pfam annotation was performed using hmmer v3.3.2 (Eddy, 1998) and the Pfam database (Finn et al., 2016). In addition, GO and Kyoto Encyclopedia of Genes and Genomes (KEGG) metabolic pathway enrichment analyses were also performed.

Protein sequences for predicted genes were annotated via alignment to functional databases such as the Pathogen-Host Interaction Factor Database (PHI) (Winnenburg et al., 2006) and the Transporter Taxonomy Database (TCDB) (Saier et al., 2006). In addition, functional annotation of carbohydrate-related enzymes based on the Carbohydrate-Associated Enzyme Database (CAZy) (Cantarel et al., 2009) was performed using hmmer v3.3.2 (Eddy, 1998).

Signal peptides are short peptide chains (usually 5–30 amino acids in length) that guide the transfer of newly synthesized proteins to the secretory pathway. The protein sequences of all predicted genes were analyzed using SignalP v4.0 (Petersen et al., 2011) and tmhmm v2.0 (Krogh et al., 2001) to identify proteins containing signal peptides and/or transmembrane helices (i.e., transmembrane proteins), respectively. To verify the accuracy of the annotated genes obtained by bioinformatics prediction, two highly expressed genes that are both secreted and effector proteins were selected for sequencing. We designed primers (Supplementary Table 2) based on the gene sequences, extracted RNA from *V. dahliae* and reverse transcribed it into cDNA. cDNA was then used as a template for gene amplification and sanger sequencing.

### Transcriptome sequencing and analysis

Three varieties of cotton with varying degrees of disease resistance were selected for transcriptomic analysis: the susceptible variety Jimian11 (JM11), the resistant variety Zhongzhimian2 (ZZM2), and Zhongmiansuo24 (ZM24). Seedlings from each strain were grown in the greenhouse for 15 days and transferred into *V. dahliae* broth upon emergence of the first true leaf (Li et al., 2021). Seedling roots were sampled at 0, 6, 12, and 24 h after transfer into *V. dahliae* broth (The time point of 0 h represented that the roots had not yet been inoculated with *V. dahliae*, and we sampled and sequenced the roots and *V. dahliae* separately). Root samples from two seedlings were combined to form a single sample, and there were three biological replicates for each strain. Root samples were sent to Beijing Biomarker Company for transcriptome sequencing.

The previously published TM-1 genome (Yang et al., 2019) and the newly assembled *V. dahliae* VD991 genome (obtained in this study) were used as reference genome. Transcriptome sequencing data were mapped to the reference genome, and count matrices and FPKM (Fragments Per Kilobase Million) values were obtained using previously described methods (Ding et al., 2019). The values from roots and *V. dahliae* separately at 0 h were combined as a control group. Differentially expressed genes (DEGs) were identified as those with a *P*-value < 0.05 and | log2-FoldChange| > 1.2. Gene ontology (Ashburner et al., 2000) and KEGG (Kanehisa et al., 2004) enrichment analyses were performed using the R-package “clusterProfiler”. In addition, a weighted gene co-expression network analysis (WGCNA) was performed using the R package “WGCNA” (Langfelder and Horvath, 2008; Duan et al., 2022), with the FPKM values as input, as described previously (Schurack et al., 2021).

### Whole-genome resequencing analysis

Eighty-seven *V. dahliae* resequencing datasets were downloaded from the NCBI SRA database^3^; the *V. dahliae* VD991 genome, newly assembled in this study, was then used as the reference genome for resequencing analysis (Yang et al., 2019). After calling SNPs, samples were grouped based on the results of a principal components analysis (PCA), population structure analysis and phylogenetic analysis. The population fixation index (F*_*ST*_*) was calculated (based on the grouping results) using vcftools v0.1.13 (Danecek et al., 2011).

## Results

### Genome sequencing, assembly, and annotation

**FIGURE 2:**
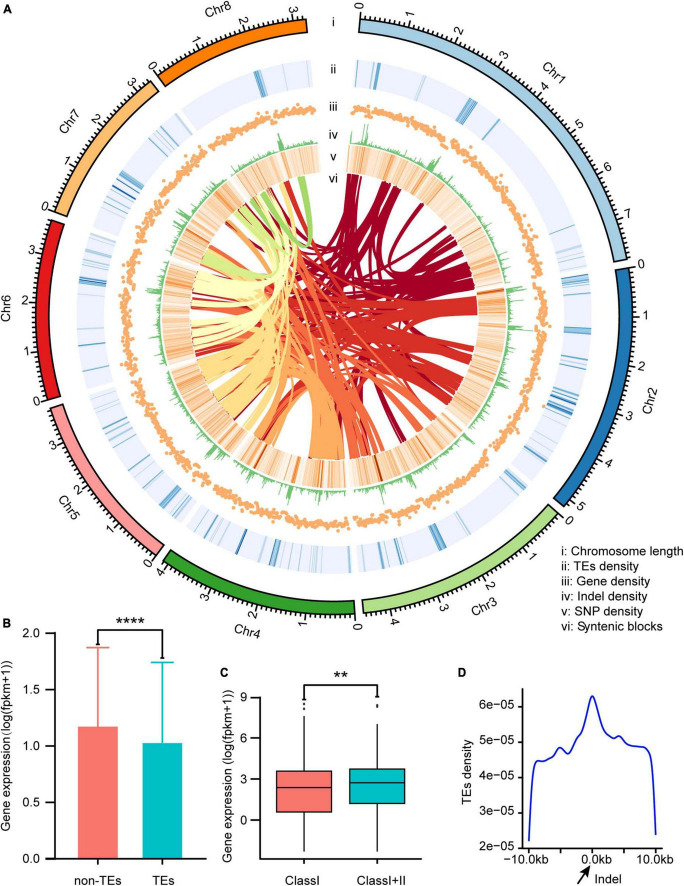
Characterization of the *Verticillium dahliae* VD991 genome. **(A)** i: length of each chromosome; ii: transposable element (TE) density in each chromosome; iii: gene density in each chromosome; iv: indel density in each chromosome; v: SNP density in each chromosome; vi: syntenic blocks in each chromosome. **(B)** The gene expression levels in the 10 kb region near TEs and not near TEs. ****P < 0.0001. **(C)** The effect of different types of TEs (Class I represents genes near Class I TEs only and Class I+II represents genes near Class I and Class II TEs) on gene expression. **P < 0.01. **(D)** The distribution of TEs in the region near the indel.

A total of 5.09 Gb of raw reads were obtained from Nanopore sequencing of *V. dahliae* VD991. After removing adapters, short fragments, and low-quality data, a total of 4.92 Gb (∼137.4 ×) of clean reads were obtained for use in whole genome assembly. The final assembled genome for *V. dahliae* VD991 consisted of nine scaffolds and a scaffold N50 length of 4,119,679 bp, with the longest scaffold having a length of 7,830,508 bp and a GC content of 53.41%. Roughly 35.77 Mb of sequence data were anchored onto eight pseudochromosomes, with 99.92% of the sequences oriented (Table 1 and Figure 2A).

**TABLE 1:** Summary statistics of *Verticillium dahliae* VD991 assembly genome compared with that of *V. dahliae* VD.Ls17 and *V. dahliae* VDJR2.

Genomic feature	VD991	VD.Ls17	VDJR2
Length of largest scaffolds (bp)	7,830,508	5,989,981	9,275,483
Number of anchored scaffolds	9	8	8
Number of anchored chromosomes	8	8	8
N50 of scaffolds (bp)	4,119,679	5,894,008	4,086,908
Number of contigs	13	/	/
GC content (%)	53.41	53.99	53.88
Total length of pseudomolecules (Mb)	35.77	35.97	36.15
Sequences anchored to chromosomes (%)	99.17%	/	/
Number of unanchored scaffolds (bp)	27,187	/	/
Percentage of transposable elements in genome size (%)	9.33	/	/
Average gene length (bp)	2,142.10	/	/
Average exon number	2.94	/	/
Average exon length (bp)	658.45	/	/
Average CDS length (bp)	518.07	/	/
Number of annotated genes	10,455	/	/
Complete prediction BUSCO	98.62%	/	/

Assembly completeness was assessed by alignment with Illumina reads and BUSCO analysis. More than 99.17% of Illumina reads mapped properly to the new assembly. Furthermore, 98.62% of 290 core conserved genes (from the fungi_odb9 database) were classified as complete in the BUSCO analysis.

Roughly a tenth (9.33%) of the assembled genome was classified as repetitive sequences (Supplementary Table 3). Class I transposable elements (TEs) constituted the predominant repeat type, accounting for 8.77% of the total genome length. A total of 10,455 genes were predicted (Supplementary Table 4) by combined *de novo*, homologous protein-based, and transcriptome-based prediction; of these, 10,441 (99.86%) genes were supported by both homologous protein-based and transcriptome-based predictions, suggesting these are well-supported genes (Supplementary Figure 1). The average gene length was 2,142 bp, with an average of 2.94 exons, 1.94 introns, and 2.87 CDS per gene. Prediction results for non-coding RNA identified 125 rRNAs, 247 tRNAs, and 36 additional unclassified non-coding RNAs. The secreted and effector proteins are considered important pathogenic factors of *V. dahliae*. In this study, 854 secreted proteins (Supplementary Table 5) and 128 effector proteins (Supplementary Table 6) were predicted. To validate the accuracy of the gene prediction, cDNA amplification and sequencing were performed on the predicted effector and secretory protein genes, and the amplified gene sequences were found to be consistent with the predicted sequences (Supplementary Figures 2, 3), confirming the accuracy of our gene prediction results.

### Effects of TEs on the genome of *Verticillium dahliae*

Gene expression levels were significantly lower in the 10-kb region surrounding each TE vs. other regions (Figure 2B). To assess how TE type affected gene expression, genes located in the vicinity of TEs were divided into those near Class I TEs only and those near Class I and Class II TEs. Genes affected only by Class I TEs showed significantly lower expression as compared to genes affected by both Class I and Class II TEs (Figure 2C). Thus, Class I TEs might suppress gene expression, while Class II TEs counteract this effect (to some extent). Transposable elements are an important source of mutations and genetic polymorphism. Many TE families are still actively transposable, and this process is highly mutagenic. In animals, plants, and microorganisms, many mutations (and the resulting phenotypic variation) are caused by transposition of these elements (Bourque et al., 2018). By calculating the density of TEs near indels (Figure 2A), more TEs were found in the vicinity of indels vs. other regions, and the density of TEs increased as the distance to the indel decreased (Figure 2D).

### Evolutionary analysis of the *Verticillium dahliae* genome

**FIGURE 3:**
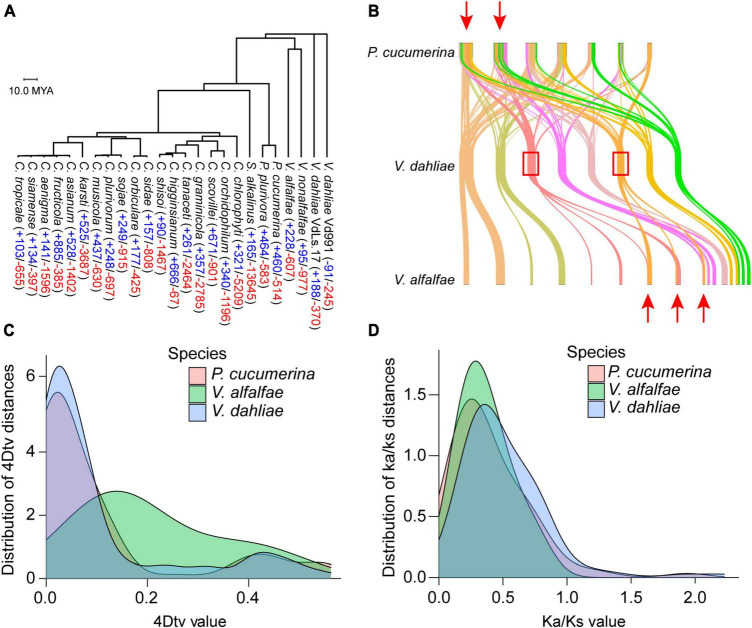
Phylogenetic and evolutionary analysis of the *Verticillium dahliae* VD991 genome. **(A)** Phylogenetic tree and gene family contraction (red) and expansion (blue) results for closely related species of *Verticillium dahliae*. **(B)** Many collinear blocks were found when comparing either the *Plectosphaerella cucumerina* or *Verticillium alfalfae* genome with the *Verticillium dahliae* genome. **(C,D)** 4DTV and ka/ks analyses revealed that the *Verticillium dahliae* genome may have undergone one WGD event.

The genomes of 25 closely related species to *V. dahliae*, including 18 *Colletotrichum* species, two *Plectosphaerella* (ascomycetes), one *Sodiomyces* and *Verticillium fungicola* (four genomes), were used to construct a phylogenetic tree. In the tree, *V. dahliae* diverges early within this evolutionary lineage. Computational analysis of gene family evolution (CAFE) was used to estimate the number of gene families that have experienced historical expansion or contraction; 53 gene families were found to have expanded and 285 gene families to have contracted in *V. dahliae* (Figure 3A). A collinearity analysis revealed that the genomes of *Plectosphaerella cucumerina* and *Verticillium alfalfae* partially overlapped with the genome of *V. dahliae*, suggesting that a whole genome duplication (WGD) event occurred in *V. dahliae* (Figure 3B). Searching for further genome duplication events, both *V. dahliae* and *Verticillium alfalfae* showed peaks at 4DTV = 0.05 (Figure 3C), and this finding was further supported by the Ka/Ks analysis (Figure 3D).

### Identification of pathogenicity-related genes in *Verticillium dahliae*

**FIGURE 4:**
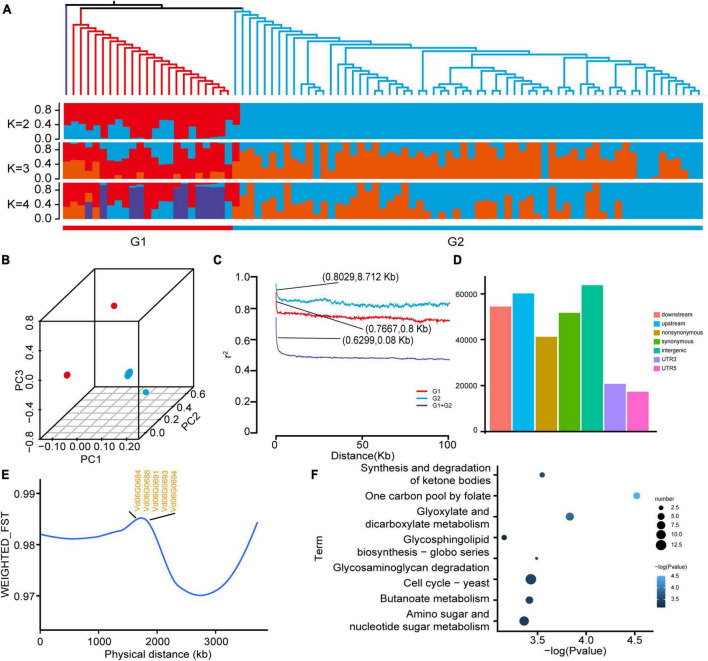
Resequencing analysis of *Verticillium dahliae* populations. **(A)** Phylogenetic tree and population structure analysis of *Verticillium dahliae* populations showed that all populations could be divided into a low-toxicity group (G1) and a high-toxicity group (G2). **(B)** PCA analysis of *Verticillium dahliae* populations was consistent with the phylogenetic tree and population structure. **(C)** Genome-wide average LD decay estimated from accessions G1, G2, and G1 + G2. **(D)** Genome-wide SNP annotation results. **(E)** Population divergence (F*_*ST*_*) among the two groups showed only partial results. **(F)** KEGG enrichment results of genes within the top 5% F*_*ST*_* values.

Using 87 *V. dahliae* genomes downloaded from the NCBI database, including both deciduous and non-deciduous types, pathogenicity-related genes were explored for *V. dahliae*: 302,949 single nucleotide polymorphisms (SNP) were identified. Phylogenetic and structural analyses based on the SNP data divided the 87 accessions into two subgroups (Figure 4A), and this division was further supported by the PCA (Figure 4B). Linkage disequilibrium (LD) analysis was used to quantify the genetic diversity within populations. Linkage disequilibrium decayed more slowly in the high-toxicity population (G2) than in the low-toxicity population (G1), indicating less genetic diversity in the high-toxicity population (Figure 4C).

Most SNPs were located in intergenic regions, suggesting they do not directly affect gene structure (Figure 4D). *V. dahliae* VD991 as a reference genome is a highly virulent strain. SNP density was measured for both subgroups (see above), and a lower SNP density was found in the high-toxicity population (Supplementary Figure 4), further reinforcing the accuracy of the grouping. To identify pathogenicity-related genes in *V. dahliae*, F*_*ST*_* values were calculated between the high- and low-toxicity populations; selecting sites with the top 5% F*_*ST*_* values, a significant region was located on chromosome 6 (Supplementary Figure 5). Annotation of this region revealed five potentially pathogenic genes, including a glucose/galactose transporter gene (Vd06G0684, Vd06G0688, Vd06G0691, Vd06G0693, and Vd06G0694) (Figure 4E and Supplementary Table 7). In addition, a KEGG analysis of all genes located within this region found significant enrichment of a number of pathways associated with disease resistance, such as yeast cell cycling and glycosaminoglycan degradation (Figure 4F).

### WGCNA analysis of transcriptome data after inoculation with *Verticillium dahliae*

**FIGURE 5:**
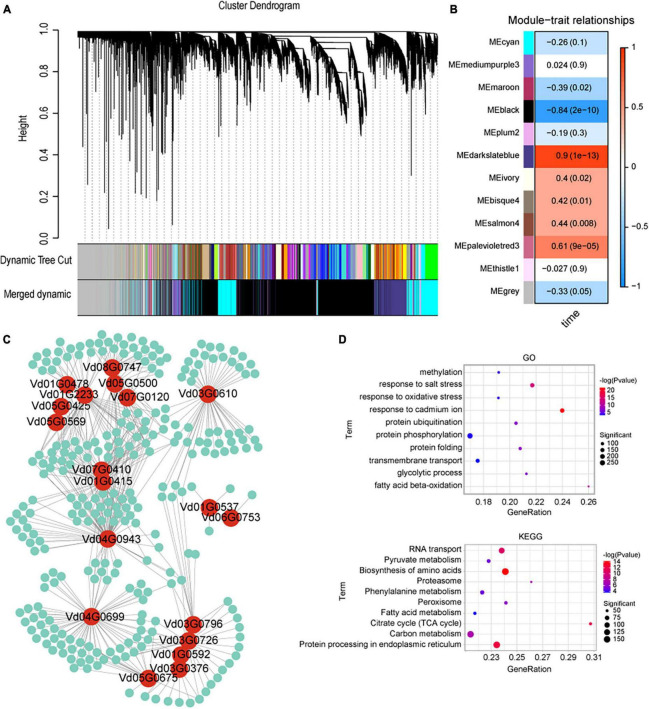
Weighted gene co-expression network analysis (WGCNA) analysis of cotton after inoculation with *Verticillium dahliae* VD991. **(A)** Gene co-expression network gene clustering number and modular cutting. Dynamic tree cut is the module divided according to clustering results. Merged dynamic is the module division of merged modules with similar expression patterns according to module similarity. The subsequent analysis was conducted by merged modules. In the case of phylogenetic trees, the vertical distance represents the distance between two nodes (between genes), while the horizontal distance is arbitrary. **(B)** Association analysis of gene co-expression network modules with physiological and biochemical traits. The horizontal axis represents different characteristics, and the vertical axis represents each module. The red lattice represents a positive correlation between the physiological traits in the module, while the blue lattice represents a negative correlation. **(C)** Visualization of interacting genes in the cyan module. Red circles represent *Verticillium dahliae* genes, green circles represent cotton genes and the lines represent interactions between them. **(D)** KEGG and GO enrichment results of all cotton genes interacting with *Verticillium dahliae* in the cyan module.

To investigate the interactions between *V. dahliae* and cotton, three cotton varieties (a susceptible variety JM11, resistant variety ZZM2, and ZM24) were inoculated with *V. dahliae* VD991 and transcriptome sequencing was performed. A total of 153.46 Gb of raw sequencing data were obtained containing 59,730 genes with large changes in expression. A WGCNA was used to investigate gene expression (in the genes with large changes in expression) at different times after *V. dahliae* inoculation, resulting in twelve genetic modules (Figures 5A, B). Effector proteins are known to play an important role in *V. dahliae* infection (Stergiopoulos and Wit, 2009; Feng et al., 2018; Wang et al., 2020); therefore, the cyan module containing the most effector protein genes was selected for subsequent analysis. By examining genes in *V. dahliae* important for interactions with cotton, pairs of interacting genes were obtained with weights greater than 0.25. A hub gene mining analysis was then performed using the MCODE package in Cytoscape; this resulted in a network containing 19 hub genes that may interact with cotton genes (Figure 5C). GO and KEGG enrichment analyses were performed of all the cotton genes interacting with *V. dahliae*. A total of 216 significantly enriched GO entries were obtained using *P* < 0.01. The top 10 entries were selected (with the smallest *P*-values, as shown in Figure 5D). In addition, the top 10 KEGG pathways were selected with *P*-values < 0.01 and gene counts (Figure 5D). In the GO and KEGG analyses, several terms and pathways were associated with resistance to *V. dahliae*, including the response to oxidative stress (GO:0006979) and phenylalanine metabolism (ko00360).

The darkslateblue module was positively correlated with *V. dahliae* post-inoculation time points (0, 6, 12, and 24 h); therefore, 5,000 gene pairs (with the highest weights) were selected from this module for analysis, resulting in a network containing 176 hub genes (Supplementary Figure 6). Ubiquitination plays an important role in plant resistance to pathogen invasion (Gao et al., 2022; Li et al., 2022). Here, 10 cotton hub genes (Gh_A08G270200, Gh_A09G093700, Gh_A09G208700, Gh_A09G238700, Gh_A10G060400, Gh_A13G194800, Gh_A13G255500, Gh_D03G157000, Gh_D03G170700, and Gh_D13G260100) were associated with ubiquitination. Two cotton hub genes (Gh_A12G006900 and Gh_D12G006300) were related to autophagy. In addition, a number of cotton genes associated with disease resistance were identified, such as genes involved in the jasmonic acid pathway (Gh_A06G223900) and catalase hydrogen peroxide (Gh_D06G205800).

### Gene expression in disease-resistant cotton after inoculation with *Verticillium dahliae*

**FIGURE 6:**
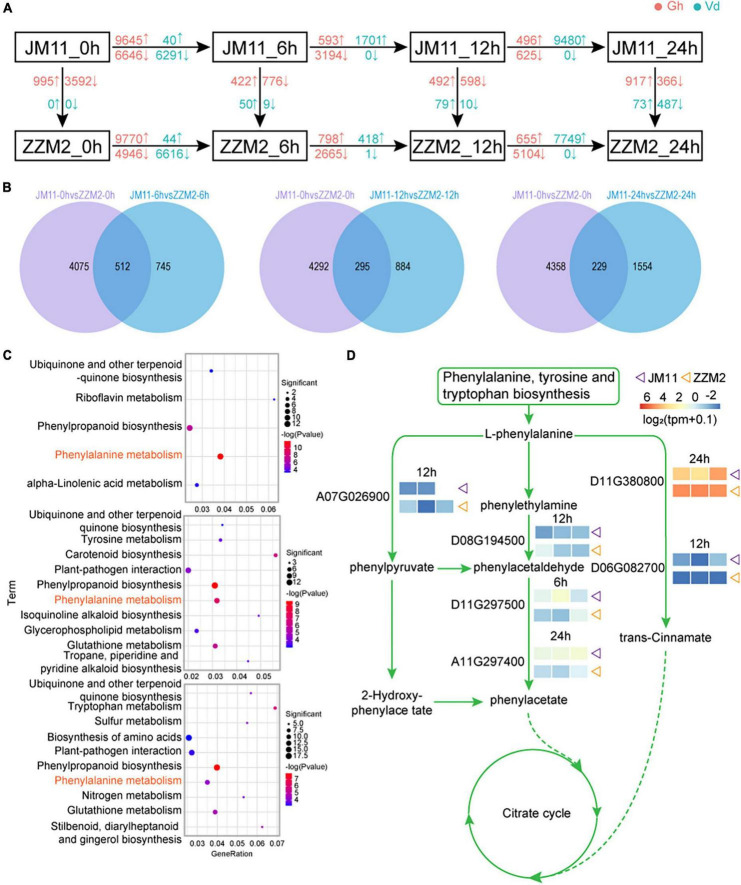
Disease resistance pathway mining of resistant and susceptible cotton. **(A)** Differential gene analysis of susceptible material JM11 and resistant material ZZM2 at different stages. Blue represents the *Verticillium dahliae* genes and red represents the cotton genes. The upward arrows indicate upregulated genes and the downward arrows indicate downregulated genes. **(B)** Venn diagram of the DEGs. **(C)** KEGG enrichment results of the common DEGs at each time point. **(D)** Visualization of the phenylalanine metabolism pathway. The expression heat map is displayed next to the corresponding gene. The purple triangle represents the susceptible material JM11 and the orange triangle represents the resistant material ZZM2.

For each post-inoculation timepoint, DEGs were identified between JM11 and ZZM2; DEGs were also identified between adjacent time points for JM11 and ZZM2 (individually). The greatest number of DEGs between JM11 and ZZM2 occurred at 6 h post-inoculation, with more up-regulated DEGs than down-regulated DEGs. By 12 h and 24 h, the number of DEGs had declined, and there were fewer up-regulated DEGs vs. down-regulated DEGs (Figure 6A). In *V. dahliae*, the number of DEGs initially increased to 6 h post-inoculation, then decreased significantly by 12 h before again increasing to a maximum at 24 h. Most of the DEGs in *V. dahliae* were down-regulated at 6 h, with almost all of the DEGs being down-regulated by 12 and 24 h (Figure 6A and Supplementary Figure 7). The common genes at these three time points and 0 h were removed (Figure 6B), leaving the remaining DEGs associated with disease resistance. A KEGG enrichment analysis of the common DEGs identified the following enriched pathways: ubiquinone and other terpenoid quinone biosynthesis, phenylpropanoid biosynthesis, and phenylalanine metabolism (Figure 6C). One of the pathways associated with disease resistance (phenylalanine metabolism) was illustrated to show the expression of DEGs in this pathway. As shown in Figure 6D, six genes were differentially expressed between JM11 and ZZM2, suggesting they may underlie the variation in disease resistance among the cotton varieties.

## Discussion

As a pathogenic fungus with multiple hosts, *V. dahliae* can infect a wide range of crops resulting in huge economic losses. However, the genetic interactions between *V. dahliae* and its hosts remain poorly understood, and more studies are still needed to better understand resistance pathways. While a large number of genomes are currently available for *V. dahliae*, these are limited by poor assembly quality and a lack of gene annotations. In this study, the genome of *V. dahliae* strain VD991 was sequenced using Nanopore and assembled into eight pseudochromosomes with a GC content of 53.41%. The genome assembly was of high quality and relatively complete (BUSCO alignment: 98.62%; Illumina reads alignment: 99.17%), with a total of 10,455 predicted genes. It has been shown previously that secretory proteins and effector proteins are important components of the toxic properties of *V. dahliae* (Duplessis et al., 2011). For example, PITG_04097, an effector protein of the oomycete *Phytophthora infestans*, is required for the inhibition of the host defense responses underlying *P. infestans’* virulence (Helm et al., 2021). Here, 854 genes involved in secreted protein synthesis and 128 genes involved in effector protein synthesis were identified in the assembled genome of *V. dahliae* strain VD991. The above results will provide useful genetic information for the study of the pathogenesis of *V. dahliae*.

Whole genome duplication events play an important role in the evolution of new species (Wu et al., 2020). It has been reported extensively in plants (Li and Barker, 2020) and has also been found in fungi (Corrochano et al., 2016). In this study, the evolution of *V. dahliae* VD991 was analyzed by comparative genomics, and one WGD event was identified in the genome of *V. dahliae* VD991. TEs are one source of mutation and genetic polymorphism that can disperse a large number of promoters, enhancers, transcription factor binding sites, insulator sequences, and repressive elements throughout the genome, thereby potentially modulating gene expression (Bourque et al., 2018). Although fungi have fewer TEs than plants, TEs still play an important role in fungal genomes (Depotter et al., 2022). For example, Urquhart et al. (2022) discovered that a large TE in *Paecilomyces variotii* could regulate its tolerance to chromium, mercury, and sodium ions. In this study, we found that the gene expression levels in the 10 kb region near the TEs were significantly lower than those not near TEs, primarily caused by Class I TEs. In addition, we detected that the density of the TE distribution was higher in the region near indels than in other regions, and the density of TEs increased with decreasing distance to the indel. Our results were similar to the findings of Viviani et al. (2021) and provide useful information for future studies of TEs in fungi.

We analyzed resequencing data of *V. dahliae* containing both deciduous and non-deciduous types. A total of 87 samples were divided into two subgroups, with the deciduous type samples contained in the high-toxicity population and the non-deciduous type samples contained in the low-toxicity population. LD results showed that the high-toxicity population had lower genetic diversity than the low-toxicity population, suggesting that the high-toxicity population may have been domesticated during evolution, resulting in a reduction in genetic diversity and increasing their virulence. We calculated F*_*ST*_* values between the high-toxicity and low-toxicity populations and identified five genes including one glucose/galactose transporter gene (Vd06G0688) that are potential pathogenic genes in *V. dahliae*. The process of invasion in cotton by *V. dahliae* first requires the destruction of the cotton cell wall. Chen et al. (2016) demonstrated that knocking out the cellulose degradation gene of *V. dahliae* reduced its ability to disrupt the cell wall of cotton, thereby reducing its virulence. The expression of the glucose/galactose transporter gene resulted in the degradation of cellulose, thereby disrupting the cell wall structure, and we hypothesized that this could be related to the pathogenicity of *V. dahliae*. In addition, KEGG analysis significantly enriched many pathways associated with disease resistance, such as yeast cell cycle and glycosaminoglycan degradation pathways (Shaban et al., 2018).

The cyan module contained 19 hub genes, all of which were found to be genes of *V. dahliae* based on the gene interaction network between *V. dahliae* and cotton. Of these, 18 were predicted to be secreted protein genes, and beta-xylosidase (Vd08G0747), carbonate dehydratase (Vd03G0796), and cutinase (Vd01G2233) were among those associated with cell wall degrading enzymes and previously reported to be related to *V. dahliae* pathogenicity (Tzima et al., 2011; Chen et al., 2016; Yang et al., 2018). Additionally, cotton genes in this module were significantly enriched with oxidative stress response terms and phenylalanine metabolism pathways, which are associated with disease resistance (McFadden et al., 2001; Ahuja et al., 2012). In the darkslateblue module, two cotton hub genes related to autophagy were identified. Autophagy has been found to increase a plant’s resistance to pathogens (Zhang B. et al., 2021). Furthermore, the analysis also highlighted cotton genes associated with disease resistance, such as those involved in the jasmonic acid pathway (Gh_A06G223900) (Liu et al., 2019) and catalase hydrogen peroxide (Gh_D06G205800) (You et al., 2022). These results demonstrate the interaction between cotton and *V. dahliae* genes and provide a reference for studying disease resistance in cotton and the pathogenesis of *V. dahliae*.

Transcriptomic analysis showed that within 6 h of inoculation with *V. dahliae*, there was a strong defensive response in cotton, with a large number of DEGs significantly upregulated, while most DEGs in *V. dahliae* were downregulated. After 6 h, a large number of DEGs in cotton were significantly downregulated, while all DEGs in *V. dahliae* were upregulated. The above results indicated that *V. dahliae* was at a disadvantage at the initial stage of inoculation with *V. dahliae* in cotton and gained an advantage after 6 h. We performed GO and KEGG enrichment analysis for all cotton genes interacting with *V. dahliae* and found several terms and pathways associated with resistance to *V. dahliae* infection, including response to oxidative stress and phenylalanine metabolism. This is consistent with previous reports that the accumulation of reactive oxygen species and phenylalanine are related to resistance to Verticillium wilt in cotton (Zhang et al., 2022). These results may be key factors contributing to the differences in disease resistance in different strains of cotton.

## Conclusion

In summary, we have sequenced and assembled a high quality genome of *V. dahliae* strain VD991 and provided a relatively complete annotation of the genome. The genes causing the differences in toxicity in *V. dahliae* VD991 were identified by resequencing analysis. We investigated the interaction between cotton and *V. dahliae* and identified many genes and pathways associated with cotton disease resistance and *V. dahliae* pathogenesis through transcriptome sequencing. These results will provide new insights into *V. dahliae* pathogenic mechanisms and contribute to the cultivation of cotton varieties resistant to Verticillium wilt.

## Data availability statement

The datasets presented in this study can be found in online repositories. The names of the repository/repositories and accession number(s) can be found below: https://www.ncbi.nlm.nih.gov/, PRJNA939821.

## Author contributions

ZY conceived and designed the research. JY performed the bioinformatics, data analysis, and wrote the manuscript. LL prepared the mRNA-sequencing samples and data. LY, RL, and CG helped in the bioinformatics analysis. LF participated in the text proofreading work. All authors contributed to the article and approved the submitted version.

## Footnotes

1
https://www.ncbi.nlm.nih.gov/genome/browse/#!/eukaryotes/832/

2
https://github.com/rrwick/Filtlong

3
https://www.ncbi.nlm.nih.gov/sra

## Supplementary material

Supplementary Figure 1 Venn diagram of the genes predicted by *de novo* prediction, homologous protein-based prediction, and transcriptome-based prediction.

Supplementary Figure 2 Verification results for the Vd01G0478 gene.

Supplementary Figure 3 Verification results for the Vd03G0726 gene.

Supplementary Figure 4 SNP density of *Verticillium dahliae* populations. **(A)** SNP density of high-toxicity group. **(B)** SNP density of low-toxicity group. **(C)** SNP number of high-toxicity and low-toxicity groups.

Supplementary Figure 5 A histogram of F*_*ST*_* in each chromosome.

Supplementary Figure 6 The network diagram was generated using 5000 pairs of genes with the highest weight in the darkslateblue module. The orange dots represent the genes in the hub subnetwork, and the others represent the genes in the non-subnetwork.

Supplementary Figure 7 The expression of DEGs is shown for susceptible material JM11 and resistant material ZZM2 at different stages.
