# Supplementary material for: High-quality genome assembly of Verticillium dahliae VD991 allows for screening and validation of pathogenic genes
Source: Front Microbiol. 2023 May 31;14:1177078. doi: 10.3389/fmicb.2023.1177078 (PMC10289290; doi:10.3389/fmicb.2023.1177078)
Supplement: Supplementary file 1 [file Table_1.docx]

**Table S1.** The situation of published genomes.

| Strain | Level | Size (Mb) | GC% | Scaffolds | Sequencing method | Gene number |
| --- | --- | --- | --- | --- | --- | --- |
| VdLs.17 | Scaffold | 33.90 | 55.60 | 55 | / | 10,535 |
| VdLs.17 | Complete | 35.97 | 53.99 | 8 | PacBio | / |
| JR2 | Complete | 36.15 | 53.88 | 8 | PacBio | / |
| Getta Getta | Chromosome | 33.30 | 55.41 | 277 | Illumina HiSeq | 8,785 |
| Gwydir1A3 | Chromosome | 31.97 | 55.62 | 163 | Illumina HiSeq | 8,654 |
| S011 | Scaffold | 37.26 | 53.40 | 9 | Illumina HiSeq; PacBio Sequel | / |
| S023 | Contig | 35.31 | 53.70 | 13 | Illumina HiSeq; PacBio Sequel | / |
| VdC07 | Contig | 34.83 | 53.80 | 26 | Oxford Nanopore MinION | / |
| CQ2 | Contig | 35.76 | 53.30 | 16 | PacBio | / |
| XJ592 | Contig | 35.67 | 53.28 | 12 | PacBio RSII; Illumina HiSeq | / |
| VdLs16 | Scaffold | 36.09 | 53.80 | 14 | PacBio; Illumina | / |
| TO22 | Contig | 35.18 | 53.90 | 14 | PacBio Sequel | / |
| GF1300 | Contig | 34.72 | 53.80 | 11 | PacBio Sequel | / |
| XJ511 | Contig | 34.90 | 54.00 | 11 | PacBio RSII; Illumina HiSeq | / |
| VdB09 | Contig | 34.64 | 53.80 | 16 | Oxford Nanopore MinION | / |
| Ud1-4-1 | Contig | 35.23 | 53.90 | 17 | PacBio Sequel | / |
| DAR82592 | Contig | 35.78 | 53.10 | 28 | Oxford Nanopore MinION; Illumina MiSeq | / |
| DAR83143 | Contig | 35.56 | 53.30 | 22 | Oxford Nanopore MinION; Illumina MiSeq | / |
| 85S | Contig | 35.89 | 53.60 | 39 | PacBio | / |
| GF1192 | Contig | 34.85 | 53.80 | 20 | PacBio Sequel | / |
| HoMCLT | Contig | 33.94 | 54.60 | 18 | PacBio Sequel | / |
| Gf-Cb5 | Contig | 35.31 | 53.60 | 18 | PacBio Sequel | / |
| DAR83175 | Contig | 35.57 | 53.20 | 41 | Oxford Nanopore MinION; Illumina MiSeq | / |
| MPI-CAGE-AT-0001 | Contig | 40.17 | 53.50 | 186 | PacBio SEQUEL | 12,591 |
| 12008 | Contig | 35.06 | 54.60 | 103 | PacBio; Illumina MiSeq | 10,485 |
| Vd991 | Scaffold | 34.71 | 54.50 | 167 | PacBio; Illumina MiSeq | / |
| WCS 071 | Scaffold | 32.51 | 55.60 | 643 | Illumina HiSeq | / |
| 12161 | Scaffold | 32.86 | 55.70 | 1238 | Illumina MiSeq | 9,933 |
| 12158 | Scaffold | 32.56 | 55.90 | 1155 | Illumina MiSeq | 9,925 |
| Vd39 | Scaffold | 35.88 | 53.40 | 1578 | Illumina | / |
| DAR82597 | Contig | 32.54 | 56.00 | 1566 | IonTorrent | / |
| 12251 | Scaffold | 33.03 | 56.00 | 1483 | Illumina MiSeq | 10,294 |
| DAR83135 | Contig | 34.37 | 54.00 | 1650 | IonTorrent | / |
| VanDijk | Scaffold | 33.14 | 54.60 | 1768 | Illumina | / |
| JKG8 | Scaffold | 33.82 | 54.40 | 1839 | Illumina | / |
| 2009-605 | Scaffold | 34.06 | 54.60 | 1930 | Illumina | / |
| ST16.01 | Scaffold | 34.19 | 54.80 | 1820 | Illumina | / |
| 12253 | Scaffold | 32.36 | 56.40 | 1382 | Illumina MiSeq | 10,330 |
| V152 | Scaffold | 33.94 | 54.20 | 2530 | Illumina | / |
| V52 | Scaffold | 33.51 | 54.30 | 3417 | Illumina | / |
| 463 | Scaffold | 34.00 | 53.30 | 4182 | Illumina | / |
| VDG2 | Scaffold | 34.17 | 54.90 | 650 | Illumina GAIIx | 9,986 |
| VDG1 | Scaffold | 32.46 | 55.60 | 510 | Illumina GAIIx | 9,795 |
