# Supplementary material for: High-quality genome assembly of Verticillium dahliae VD991 allows for screening and validation of pathogenic genes
Source: Front Microbiol. 2023 May 31;14:1177078. doi: 10.3389/fmicb.2023.1177078 (PMC10289290; doi:10.3389/fmicb.2023.1177078)
Supplement: Supplementary file 2 [file Table_2.docx]

**Table S2.** Primer sequences of gene verification.

| GeneID | F | R |
| --- | --- | --- |
| Vd01G0478 | GGCGAGGCGTGGTTATACCT | TTATTTGGTGTCGTCTCGGATGA |
| Vd03G0726 | ATGTCGGCGTACACAACC | TTAGCTGTGGAATGCCTTG |
