# Supplementary material for: High-quality genome assembly of Verticillium dahliae VD991 allows for screening and validation of pathogenic genes
Source: Front Microbiol. 2023 May 31;14:1177078. doi: 10.3389/fmicb.2023.1177078 (PMC10289290; doi:10.3389/fmicb.2023.1177078)
Supplement: Supplementary file 3 [file Table_3.docx]

**Table S3.** Predicted results for different types of repetitive sequences.

| **Type** | **Number** | **Length (bp)** | **Percentage (%)** |
| --- | --- | --- | --- |
| ClassI | 1,417 | 3,174,308 | 8.87 |
| ClassI/DIRS | 257 | 381,847 | 1.07 |
| ClassI/LINE | 67 | 53,130 | 0.15 |
| ClassI/LTR | 1 | 147 | 0 |
| ClassI/LTR/Copia | 466 | 748,035 | 2.09 |
| ClassI/LTR/Gypsy | 544 | 1,820,985 | 5.09 |
| ClassI/PLE\|LARD | 43 | 206,657 | 0.58 |
| ClassI/SINE | 1 | 63 | 0 |
| ClassI/TRIM | 21 | 5,257 | 0.01 |
| ClassI/Unknown | 17 | 35,402 | 0.1 |
| ClassII | 90 | 17,532 | 0.05 |
| ClassII/Helitron | 4 | 220 | 0 |
| ClassII/MITE | 23 | 5,937 | 0.02 |
| ClassII/TIR | 57 | 10,895 | 0.03 |
| ClassII/Unknown | 6 | 480 | 0 |
| Unknown | 357 | 148,634 | 0.42 |
| Total | 1,507 | 3,336,413 | 9.33 |
