# Supplementary material for: High-quality genome assembly of Verticillium dahliae VD991 allows for screening and validation of pathogenic genes
Source: Front Microbiol. 2023 May 31;14:1177078. doi: 10.3389/fmicb.2023.1177078 (PMC10289290; doi:10.3389/fmicb.2023.1177078)
Supplement: Supplementary file 4 [file Table_4.docx]

**Table S4.** The genes predicted by de novo prediction, homologous protein-based prediction and transcriptome-based prediction.

| **Method** | **Software** | **Species** | **Gene number** |
| --- | --- | --- | --- |
| Ab initio based | Augustus |  | 8,330 |
|  | Genscan |  | 7,834 |
|  | GeneID |  | 13,830 |
|  | GlimmerHMM |  | 8,964 |
|  | SNAP |  | 9,346 |
| RNA-seq based | PASA |  | 12,923 |
|  | TransDecoder |  | 26,924 |
| Homology based | GeMoMa | Verticillium alfalfae | 9,722 |
|  |  | Verticillium dahliae | 10,235 |
|  |  | Verticillium nonalfalfae | 9,174 |
| Integration | EVM |  | 10,455 |
