# Supplementary material for: High-quality genome assembly of Verticillium dahliae VD991 allows for screening and validation of pathogenic genes
Source: Front Microbiol. 2023 May 31;14:1177078. doi: 10.3389/fmicb.2023.1177078 (PMC10289290; doi:10.3389/fmicb.2023.1177078)
Supplement: Supplementary file 7 [file Table_7.docx]

**Table S7.** The annotation results of the pathogenic gene identified on chromosome 6.

| **Gene ID** | **Gene function** |
| --- | --- |
| Vd06G0694 | NAD-dependent histone deacetylase SIR2 |
| Vd06G0688 | glucose/galactose transporter |
| Vd06G0684 | kinesin heavy chain |
| Vd06G0691 | siderophore iron transporter mirB |
| Vd06G0693 | dual specificity protein kinase pom1 |
