# Supplementary figures and images for: High-quality genome assembly of Verticillium dahliae VD991 allows for screening and validation of pathogenic genes
Source: Front Microbiol. 2023 May 31;14:1177078. doi: 10.3389/fmicb.2023.1177078 (PMC10289290; doi:10.3389/fmicb.2023.1177078)

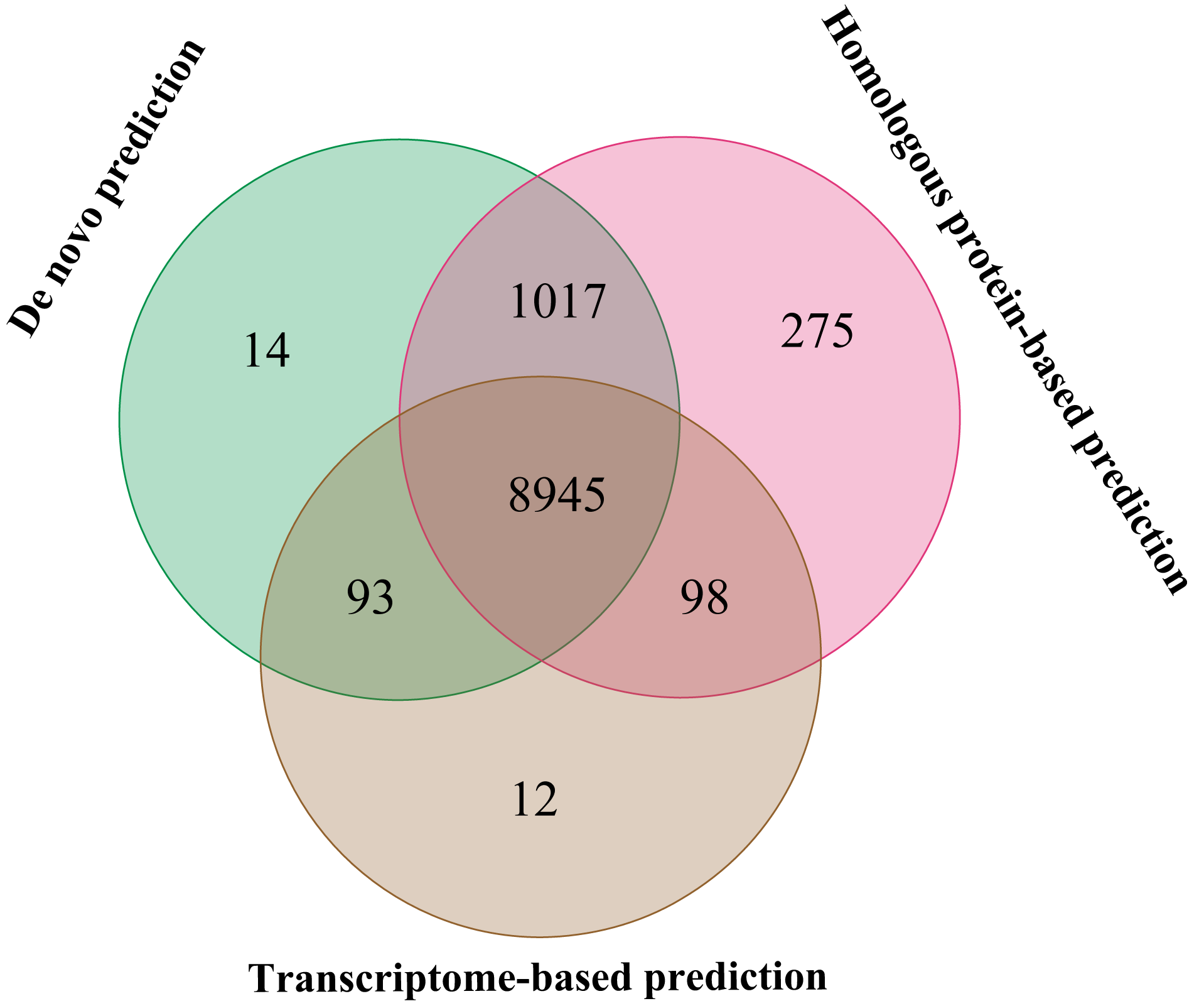

Supplement: Supplementary Figure 1 — Venn diagram of the genes predicted by de novo prediction, homologous protein-based prediction, and transcriptome-based prediction. [file Image_1.TIF]

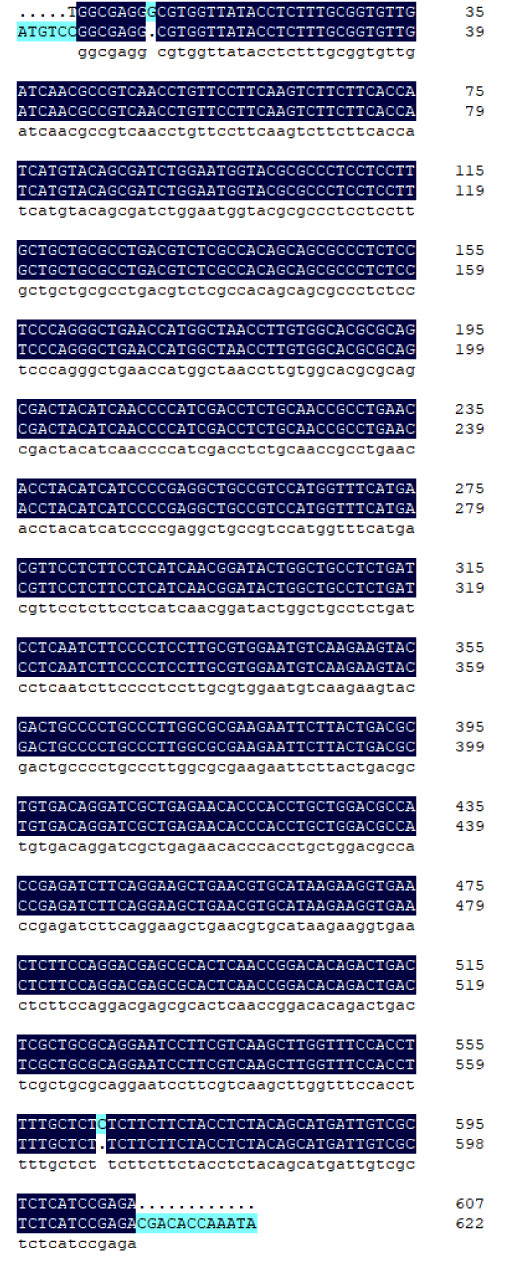

Supplement: Supplementary Figure 2 — Verification results for the Vd01G0478 gene. [file Image_2.TIF]

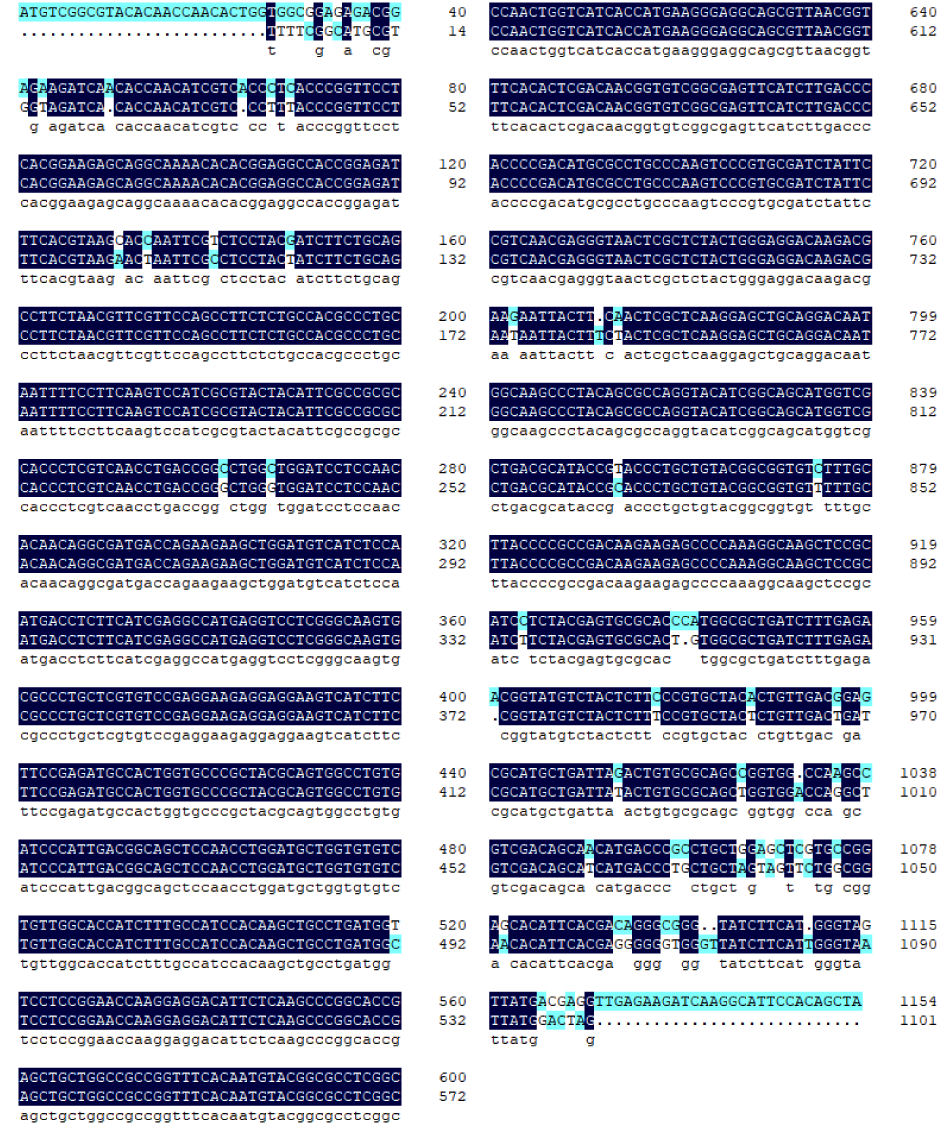

Supplement: Supplementary Figure 3 — Verification results for the Vd03G0726 gene. [file Image_3.TIF]

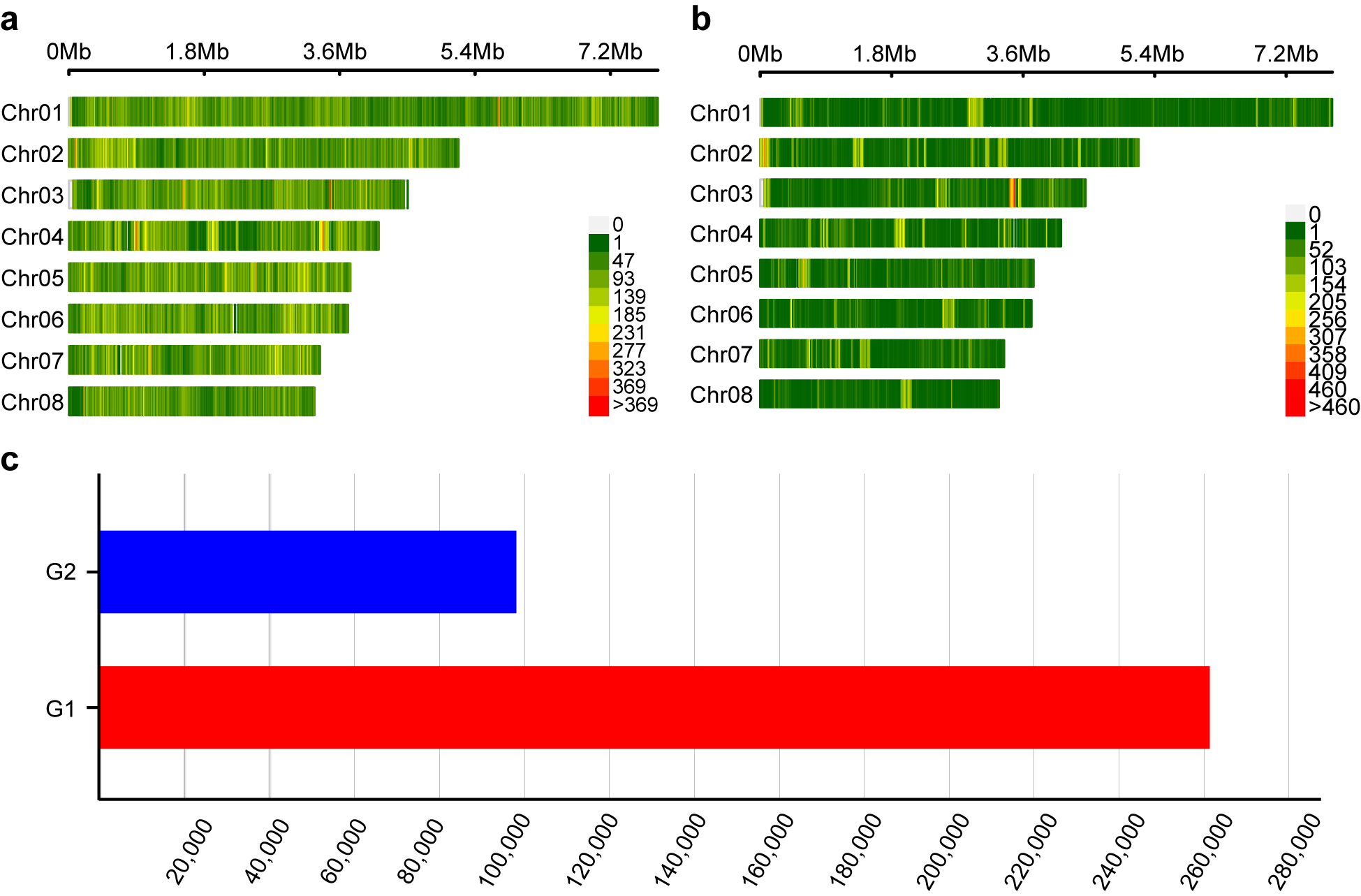

Supplement: Supplementary Figure 4 — SNP density of Verticillium dahliae populations. (A) SNP density of high-toxicity group. (B) SNP density of low-toxicity group. (C) SNP number of high-toxicity and low-toxicity groups. [file Image_4.TIF]

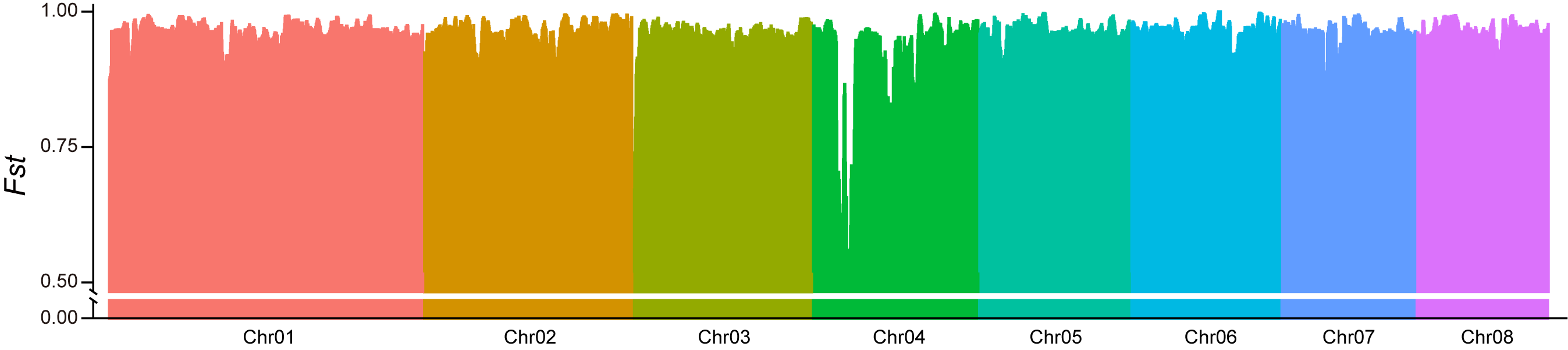

Supplement: Supplementary Figure 5 — A histogram of FST in each chromosome. [file Image_5.TIF]

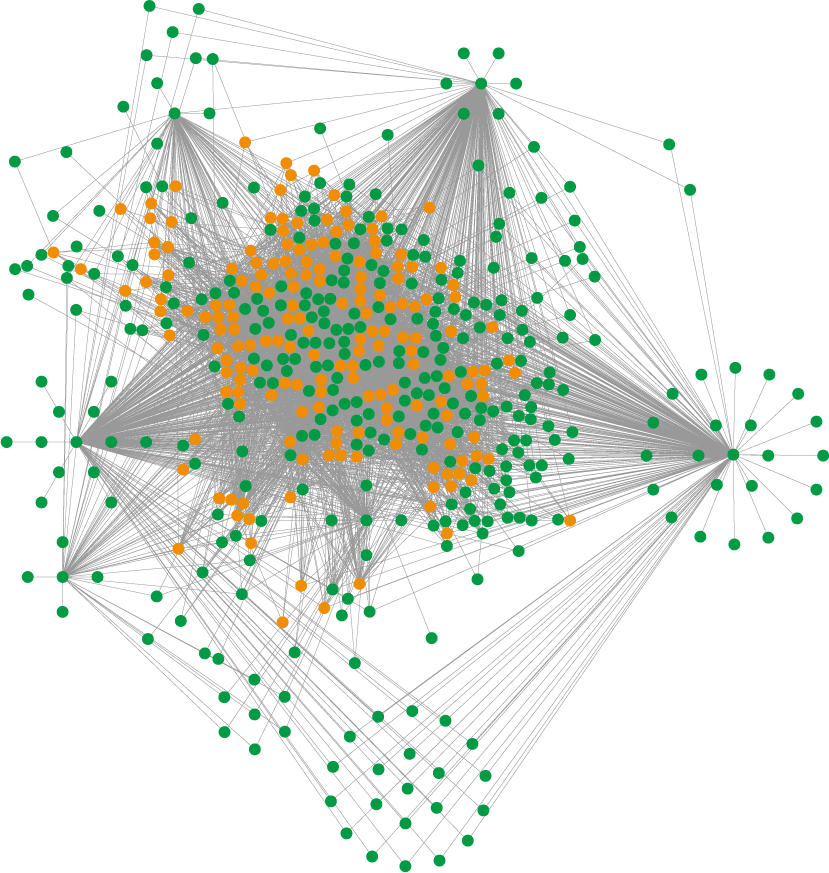

Supplement: Supplementary Figure 6 — The network diagram was generated using 5000 pairs of genes with the highest weight in the darkslateblue module. The orange dots represent the genes in the hub subnetwork, and the others represent the genes in the non-subnetwork. [file Image_6.TIF]

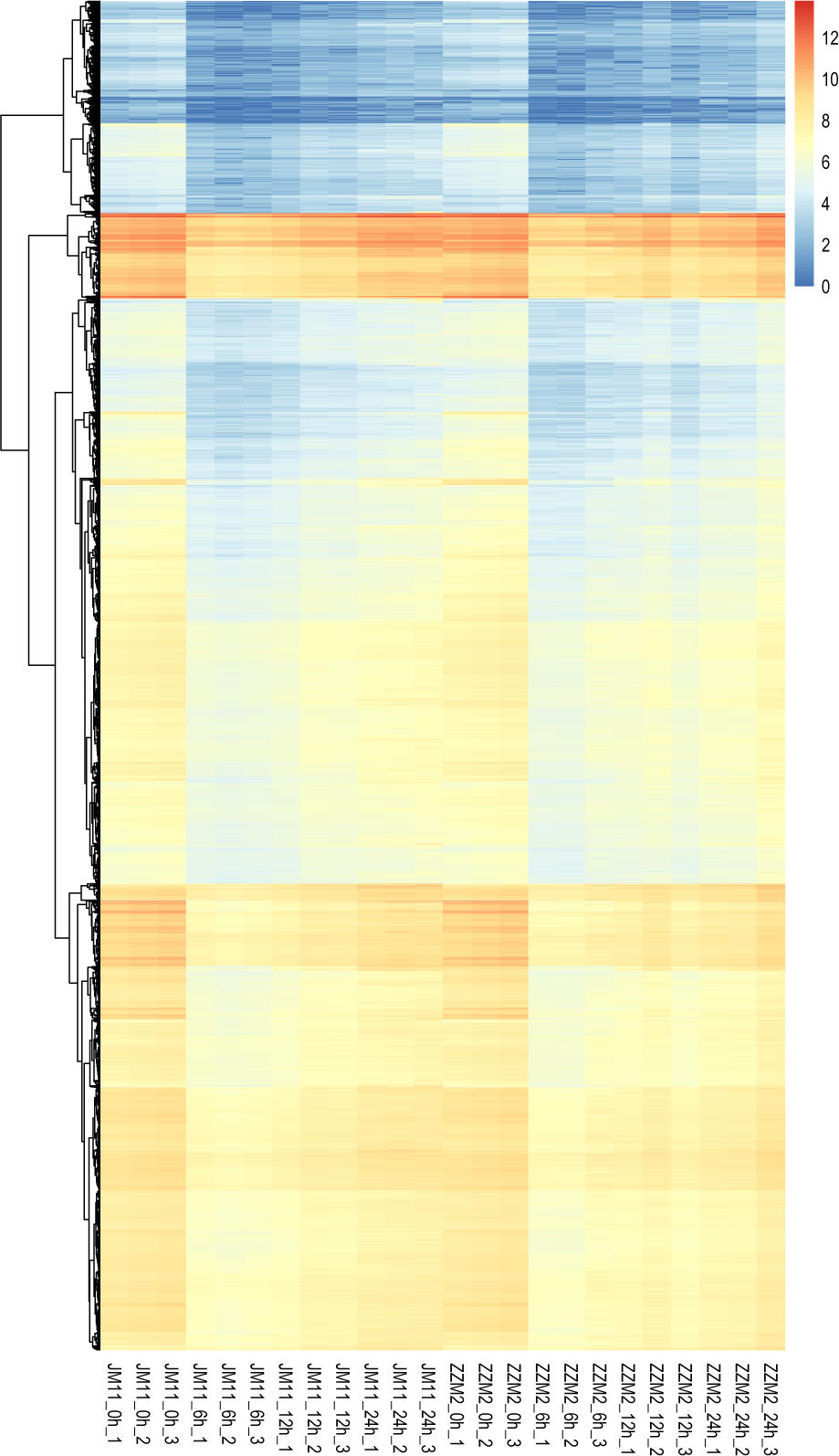

Supplement: Supplementary Figure 7 — The expression of DEGs is shown for susceptible material JM11 and resistant material ZZM2 at different stages. [file Image_7.TIF]
